# Complementing endozoochorous seed dispersal patterns by donkeys and goats in a semi-natural island ecosystem

**DOI:** 10.1186/s12898-017-0148-6

**Published:** 2017-12-19

**Authors:** Julia Tabea Treitler, Tim Drissen, Robin Stadtmann, Stefan Zerbe, Jasmin Mantilla-Contreras

**Affiliations:** 1 RG Ecology and Environmental Education, Institute of Biology and Chemistry, University of Hildesheim, Universitätsplatz 1, 31141 Hildesheim, Germany; 2 Institute of Geography, University of Hildesheim, Universitätsplatz 1, 31141 Hildesheim, Germany; 3 Faculty of Science and Technology, Free University of Bozen-Bolzano, Piazza Università 5, 39100 Bozen, Italy

**Keywords:** Grazing, Germination experiment, Plant functional groups, Mediterranean region

## Abstract

**Background:**

Endozoochory is, in grazing systems, a substantial vector for seed dispersal. It can play an important role in vegetation dynamics, especially in colonization processes through seed input on the vegetation and on the soil seed bank. We investigated the endozoochorous seed input of donkeys and goats on a semi-natural island ecosystem in the Mediterranean. Through germination experiments, we assessed the viable seed content of the dung of these grazing animals to estimate their suitability and efficiency for seed dispersal of the vegetation types of the island.

**Results:**

We show different dispersal patterns of donkeys and goats. Goats disperse a high number of diaspores from shrubs while donkeys disperse more diaspores of grasses. In addition, goats disperse plants of greater growth height and donkeys plants of shorter height. These dispersal patterns are in accordance with the vegetation types of which donkeys and goats disperse indicator species. Both, donkeys and goats, feed on and disperse species of the vegetation types, open grassland and temporarily wet grassland. In addition, goats feed on and disperse diagnostic species of the semi-open maquis and preforest formations.

**Conclusions:**

Overall, our results show that donkeys and goats are complementing each other in their endozoochorous seed dispersal potential. This emphasizes the importance of both grazing animals for the vegetation dynamics of the semi-natural island ecosystem. Therefore, the adaption of the goat management to a traditional land management based on directed transhumance might maintain and enrich vegetation types.

**Electronic supplementary material:**

The online version of this article (10.1186/s12898-017-0148-6) contains supplementary material, which is available to authorized users.

## Background

Seed dispersal and re-colonisation processes are decisive factors for the regulation of the community structure of plants [1]. Due to intraspecific competition with the mother plant and conspecific seedlings, dispersal plays an important role for the successful establishment of the diaspore [2]. Considering that the composition of the vegetation, dispersal, and colonisation are based on natural fluctuations essential for both stable (e.g. late-successional) as well as unstable (e.g. early-successional) habitats [3], the dispersal of diaspores enables the conservation of fragmented and permanently changing populations in a consolidated vegetation [4].

Wind (anemochory), streaming water (hydrochory) or animals (zoochory) disperse diaspores over long distances [5–7]. In grasslands, endozoochory was shown to be an effective dispersal process [8]. In particular, in the Mediterranean region endozoochory by herbivores plays an important role [8, 9] as has been shown that, in grazing systems, on average, 740 seeds per square metre are dispersed endozoochorously [9]. In addition, the long history of livestock farming has promoted the adaptability of plants to grazing [10]. Through their dung animals, like sheep and hare, disperse a high number of plant species however the effect of the passage through the gut can vary between animal species and thus can have consequences on the germination and survival of the seed [11–13]. Nevertheless, through seed dispersal animals increase species richness and spatial homogeneity through an intensification of the intra- and intercommunity seed flow [14]. This seed input on the vegetation and to the soil seed bank can be of high importance for colonization processes [9, 15] and regeneration [14, 16]. Success of colonization and regeneration dominates in early-successional habitats and in most forests [17] and depends, for example, on local characteristics like light and ground conditions [18] created by disturbance [16], and safe sites [19].

Grazing is considered one of the most important factors which alters natural processes by directly or indirectly affecting ecosystems [20] thus it might be a key factor for conservation and maintenance of biodiversity. Besides dispersal of diaspores and the creation of safe sites for plant germination [21], grazing animals affect the structure and composition of plant communities by disturbance and suppression of certain species susceptible to grazing [20, 22, 23]. Grazing, thus, has an impact on the establishment, growth and survival rate of species, and influences the abiotic conditions of the ecosystem [24]. In addition to plant defoliation, grazing animals alter the competitive interactions of plants [25, 26]. Furthermore, grazing animals compact the soil through trampling [27, 28], and with their faeces they provide high nutrient concentrations [29–31] and thus influence the conditions of germination. However, intensive grazing or the introduction of alien ungulates may threaten the biodiversity [32] and the survival of endangered plant and animal species, particularly in isolated ecosystems like islands [33].

The Italian island of Sardinia is one of the regions with the highest biodiversity, especially considering the flora [34, 35]. In the Northwest of Sardinia on the island of Asinara (51.9 km^2^), 709 plant species have been recorded [36, 37], including 35 species endemic to Sardinia and other Western Mediterranean islands [38]. This plant diversity originates from the highly structured landscape that is: a rich topography and coastal zones, with the corresponding different soil types, and also, from the variety of grazing animals such as horses, donkeys, goats, wild boars and mouflons, which were introduced on the island for centuries [36–38].

In the Mediterranean region, goats are a productive way of using areas dominated by scrublands [39], however, under not adequate management goats can cause decline and deterioration of the vegetation [40, 41]. On the island of Asinara goats and donkeys roam wild throughout the island but goats are considered to cause a decline in biodiversity, therefore the capture of goats has been implemented to remove them from the island.

Through our study, we seek to assess the potential and contribution of donkeys and goats as endozoochorous seed dispersers in order to estimate their possible input on the vegetation and colonization processes and thus, their suitability and efficiency for seed dispersal in a semi-natural island ecosystem. Up to now, there are hardly any studies investigating the dispersal capacity of grazing animals in a complete Mediterranean island ecosystem. The knowledge about the importance of donkeys and goats for the dispersal of plants and the regulation of the plant community structures on the island is necessary to evaluate and produce viable management implementations.

As donkeys and goats differ in their feeding behaviour [42, 43], we hypothesize to see differences in the dispersal capacity. Donkeys are expected to contribute to a higher part to the distribution of grasses, while goats disperse a higher variety of growth forms (e.g. grasses, dwarf shrubs, large browse plants). Due to the phenology of plants and fodder preferences of donkeys and goats, we expect differences in their dispersal capacities with the course of the season. Consequently, we focus on the following particular research questions:

1. How do donkeys and goats differ in their endozoochorous dispersal capacity?
2. Is there a seasonal effect on the dispersal capacity of donkeys and goats?

## Methods

### Study area

Our study area is the Asinara National Park, an island with an area of 51.9 km^2^ located at the northwest of Sardinia (Italy). Asinara has a typical Mediterranean climate with a dry hot summer and a rainy season between October and April. The mean annual temperature is 17.7 °C and the mean annual precipitation is 430 mm. During the wet season, mean temperatures range between 11.0 and 19.5 °C, mean humidity is about 80% and mean precipitation about 300 mm (Osservatorio ambientale Parco Nazionale dell’ Asinara, Fornelli, Asinara 2014).

Formerly, the island hosted various prisons with prisoners working in the agriculture, cultivating crops and rearing livestock [36, 44]. After the prison closure in 1997, these animals have been roaming wild on the island. Therefore, the National Park, founded in the same year, is now inhabited by grazing animals, which have become feral, namely donkeys, horses, and goats. In addition to these grazing animals, mouflon and wild boars were introduced to the island in the 1950s [45]. There are about 330 donkeys and approximately 1400 goats on the island (Parco Nazionale dell’ Asinara 2013). Since goats are intermittently captured to reduce their number according to the management plan of the National Park and the reproduction rate of goats is quite high, the number of goats may have had some fluctuations.

During the prison time, the spread of the plant communities was determined by fire, agricultural land use and grazing [36]. Today, the vegetation is mainly characterized by a typical Mediterranean maquis (e.g. *Euphorbia dendroides, Pistacia lentiscus*) and garrigue (e.g. *Cistus monspeliensis*), however Asinara is a highly diverse island regarding plants exhibiting various vegetation types like coastal vegetation (e.g. *Centaurea horrida*, *Helichrysum italicum* subsp. *microphyllum*), grassland (e.g. *Erodium moschatum, Hordeum marinum*), shrub vegetation such as woodland and forest (e.g. *Juniperus phoenicea*, *Quercus ilex*). Due to the intensive land-use during the last decades, the area of the *Quercus ilex* forest was diminished to a small part in the North and some remnants in the southern part (0.203 km^2^, 0.40%).

### Dung sampling

Dung of donkeys and goats was collected fortnightly from the end of March until mid of August 2014. The island was evenly divided into eight sampling areas. During each sampling session, a fresh sample per animal species was collected (total number of samples for donkeys is 87 and goats 88) randomly within each of the eight sampling areas. The collected dung was dried and stored at room temperature until it was processed. This treatment resembles natural conditions, because dung dries up in the field and almost all plant species in the Mediterranean germinate during autumn [15].

### Germination experiment

The viable seed content of the dung was determined by a greenhouse germination experiment. Each sample (9 g) was crumbled and prepared for the germination in sterile soil. The samples were kept moist for a 6-month period. Experimental conditions in the greenhouse were (1) mean temperature 19.1 °C (range 11.7–35.6 °C), (2) mean relative humidity of 67% (range 25–94%) and (3) day length which was adapted to the mean day length of the island during the wet season i.e. 10 h 22 min. Light cycles were adapted by using plant luminaries (high pressure sodium vapour lamp Sirius X400, Bio Green OHG, Bischoffen-Oberweidbach, Germany; 55,000 Lumen at 1.3 m distance) with mean PAR values of 200 µmol m^−2^ s^−1^ (MQ-200, Apogee Instruments, Inc., Logan, Utah, USA). Position of pots was changed at a weekly interval to provide the same conditions for all samples. To account for external seed entry, ten pots containing only sterile soil were set up as a control for seed contamination. Germinating seedlings in these control pots were left out of the data analysis (*Salix caprea*, which does not occur on Asinara).

During the germination experiment (6 month), all seedlings were counted and identified to the lowest taxonomic group possible (species, genus or family). Seedlings were removed as soon as possible after the germination to reduce competitive effects. Then, seedlings were transferred to pots to allow them to flower and to facilitate the identification of species. When germination stagnated, dung was remixed to allow remaining seeds to germinate. Species were identified using literature [46, 47], following the nomenclature of [48]. All species were classified based on plant traits to five functional types (shrub, forb, leguminous forb, grasses, and sedges and rushes) and based on their mean growth height (taken from Pignatti [46]) to different height classes (≤ 10, ≤ 20, ≤ 40, ≤ 60, ≤ 80, ≤ 100, and > 100 cm).

### Vegetation analysis

Based on remote-sensing techniques and digital image analysis, a digital mapping of the main vegetation types of the island of Asinara was performed by using the program ERDAS IMAGINE 2015 (Hexagon Geospatial, Madison, US) with field data and high resolution satellite images to conduct a pixel-based supervised classification via maximum-likelihood algorithm. These interim results were evaluated and improved by implementing the visual interpretation of orthophotos and integrating auxiliary geodata in a geographic information system (ArcGIS Desktop 10.4.1, ESRI, Redlands, US) [49]. The digital map was used to calculate the proportional area of each vegetation type on the island. The main vegetation types of the island in physiognomy and cover are: coastal vegetation mainly at the shoreline dominated by dwarf scrubs, annual forbs, and graminoids (COA); open grassland with annual graminoids, annual forbs and legumes (GRA); temporarily wet grassland located in slight depressions and dominated by graminoids, annual forbs, and legumes (TWG); garrigue dominated by *Cistus monspeliensis* shrubs (CIS); semi-open maquis dominated by *Euphorbia dendroides* (EUP); large maquis to preforest formations with *Olea europaea* (OLI); semi-open maquis to preforest formations of *Juniperus phoenicea* (JUN); forest of *Pinus pinea* (PIN); and holm oak forest of *Quercus ilex* (QUE). Additionally, there is an intermediate vegetation type of low-growing heterogeneous grassy and herbaceous vegetation (LOW), a vegetation type dominated by *Juncus acutus* (JUC) and some areas covered by *Tamarix* spp. (TAM). Vegetation surveys were performed on 88 randomly selected study sites (10 × 10 m) representing the main vegetation types according to the physiognomic structure of the plant cover. Surveys were conducted between March and May and to record late flowering species study sites were checked again between July and August. Using a continuous percentage scale, the cover of plant species was assessed.

### Data analysis

Parametric tests (paired t tests) were used to compare donkeys and goats in the mean number of germinated seedlings, plant species, and plant functional types. Further differences between donkeys and goats in their dispersal of plant functional types and different growth heights were analysed using generalised linear mixed models (GLMMs). The plant functional type model included animal species and plant functional type as explanatory variables whereas the model of growth height contained animal species and growth height as explanatory variables. The response variable in both models was the number of seedlings. To examine seasonal influences on the dispersal by donkeys and goats, we ran further GLMMs for the number of germinated seedlings and the number of viable species, with animal species and month inserted as explanatory variables. All GLMMs were carried out using the ‘lme4’ package [50] with Poisson distribution as the data originated from counts. We tested for over-dispersion and in case the data were over-dispersed, we used negative binomial distribution. The sampling area was included in all models as a random factor. All models were checked for homogeneity of variance.

For each vegetation type, we determined diagnostic species by an indicator species analysis [51] using PC-ORD 6.22 (MjM Software, Gleneden Beach, US). The Phi coefficient was assessed as a measure of fidelity [52, 53] and the significance of the observed maximum indicator value was tested by a Monte Carlo permutation test (4999 permutations). Species with Phi values greater than 0.5 (p ≤ 0.05) were considered as diagnostic species. To analyze if diagnostic species of the main vegetation types correlate with the viable species distributed by donkeys and goats, we performed Spearman correlations with the number of germinated diagnostic species of each main vegetation type and the number of viable species of the dung of donkeys and goats. In all analyses on the number of seedlings, one sample of goat was left out because it contained 1456 seedlings with the majority of seedlings belonging to the species *Juncus acutus*. All statistical analyses were conducted using R statistical package version 3.1.3 [54].

## Results

The digital mapping of the main vegetation types of the island (Additional file 1) revealed that most of the area of the island is dominated by semi-open maquis with *Euphorbia dendroides*, garrigue with *Cistus monspeliensis* shrubs and open grassland with annual graminoids, annual forbs and legumes. Vegetation types that are preforest formations or forest (e.g. *Juniperus phoenicea* formations, *Quercus ilex* forest, *Pinus pinea* forest) cover only a minor part of the whole area (Table 1).

**Table 1:** Proportional coverage and mean species richness (± SD) of the main vegetation types

	Description	Area (%)	Species richness
EUP	Semi-open maquis dominated by *Euphorbia dendroides*	26.83	69.2 (± 14.6) (N = 15)
CIS	Garrigue dominated by *Cistus monspeliensis* shrubs	23.37	73.1 (± 10.2) (N = 10)
GRA	Open grassland with annual graminoids, annual forbs and legumes	18.58	56.1 (± 8.3) (N = 11)
NOVEG	Infrastructure, rocks	16.65	0
COA	coastal vegetation mainly at the shoreline dominated by dwarf scrubs, annual forbs and graminoids	8.86	60.9 (± 13.1) (N = 19)
TWG	Temporarily wet grassland located in slight depressions and dominated by graminoids, annual forbs and legumes	1.57	58.5 (± 15.5) (N = 10)
QUE	Holm oak forest of *Quercus ilex*	0.40	34.0 (± 6.7) (N = 5)
OLI	Large maquis to preforest formations with *Olea europaea*	0.33	66.0 (± 15.8) (N = 6)
JUN	Semi-open maquis to preforest formations of *Juniperus phoenicea*	0.33	58.1 (± 18.3) (N = 9)
PIN	Forest of *Pinus pinea*	0.04	49.7 (± 5.4) (N = 3)

**Fig. 1:**
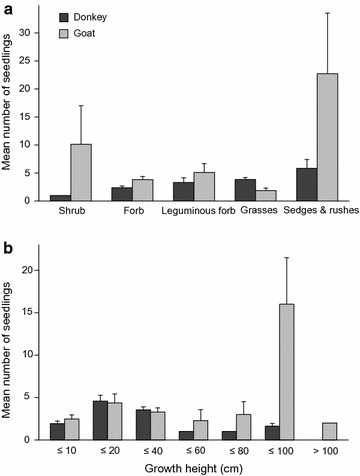
Plant functional types (**a**) and growth height (**b**) weighted by the mean number of seedlings that germinated of the dung of donkeys and goats. Error bars represent standard errors (SE)

A total of 618 seedlings of 90 plant species (83 species and 7 species groups) belonging to 17 plant families germinated in the dung samples of donkeys, while in the samples of goats 2395 seedlings of 72 plant species (69 species and 3 species groups) and 23 corresponding plant families emerged (Additional file 2). Out of the whole species pool of Asinara island (709 plant species, [36, 37], donkeys dispersed 11.7% and goats 9.7% of the occurring plant species. The mean number of germinated seedlings in the dung of donkeys (7.14 ± 1.76) and goats (10.83 ± 7.57) did not show significant differences [paired t (7) = − 1.3591, p = 0.216], however, there was a greater variability in the dung of goats. The mean number of viable plant species in the dung differed significantly between donkeys (3.75 ± 0.98) and goats (2.36 ± 0.86) [paired t (7) = 3.276, p = 0.0135]. In addition, the mean number of dispersed plant functional types [paired t (7) = 3.7886, p = 0.0068] differed significantly between donkeys (1.91 ± 0.33) and goats (1.34 ± 0.31). Donkeys and goats showed significant differences in the dispersal of plant functional types (Table 2). However, sedges and rushes were those functional types with the most seedlings in the dung for both, donkeys and goats. The samples of goats exhibited a high number of shrubs and leguminous forbs while donkeys dispersed a high number of seeds of grasses but also of leguminous forbs (Fig. 1a). In comparison, in the dung of goats significantly more seedlings of forbs and sedges and rushes germinated than in the dung of donkeys. In addition, there was a considerable trend of more shrubs that potentially can be dispersed by goats than by donkeys. In contrast, the dung of donkeys contained significantly more viable seeds of grasses than the dung of goats.

**Table 2:** GLMM on number of seedlings of plant functional types and growth heights with estimates, standard error (SE) and p value

	Estimate	SE	p value
Plant functional type			
Intercept	0.867	0.159	< 0.001***
Goat	0.474	0.222	0.032*
Grasses	0.477	0.213	0.025*
Leguminous forb	0.328	0.274	0.232
Sedges and rushes	0.896	0.231	< 0.001***
Shrub	− 0.867	0.974	0.373
Goat:grasses	− 1.190	0.394	0.002**
Goat:leguminous forb	− 0.041	0.386	0.914
Goat:sedges and rushes	0.884	0.339	0.009**
Goat:shrub	1.840	1.044	0.078.
Growth height			
Intercept	0.660	0.189	< 0.001***
Goat	0.240	0.300	0.423
GH ≤ 100	− 0.185	0.420	0.658
GH > 100	− 0.194	0.747	0.794
GH ≤ 20	0.858	0.220	< 0.001***
GH ≤ 40	0.605	0.223	0.006**
GH ≤ 60	− 0.655	0.641	0.306
GH ≤ 80	− 0.667	0.580	0.250
Goat:GH ≤ 100	2.054	0.530	< 0.001***
Goat:GH ≤ 20	− 0.302	0.347	0.385
Goat:GH ≤ 40	− 0.322	0.355	0.365
Goat:GH ≤ 60	0.574	0.774	0.458
Goat:GH ≤ 80	0.870	0.820	0.288

p value with: *** = < 0.001; ** = < 0.01; * = < 0.05;. = < 0.1; n.s. = non-significant

Donkeys and goats showed significant differences in the dispersal of plants with different growth height (Table 2). The dung of goats exhibited significantly more seedlings of greater growth height (80–100 cm). However, donkeys’ dung contained significantly more viable seeds of plants with small growth heights like the two height classes 10–20 and 20–40 cm (Fig. 1b).

**Fig. 2:**
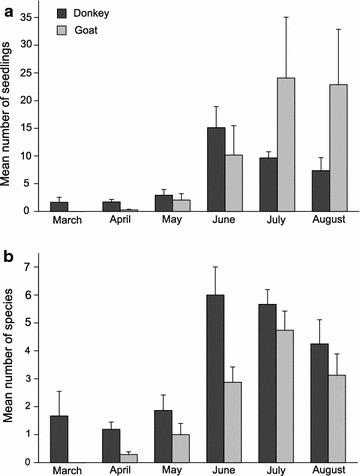
Differences during the seasonal course (March–August) between donkeys and goats in **a** the mean number of seedlings and **b** the mean number of species. Error bars represent standard errors (SE)

In the seasonal course between March and August, donkeys and goats show significant differences in the number of germinated seedlings (Table 3) in their dung. From the dung samples of the earlier months (March–June) significantly fewer seedlings germinated from the dung of goats. The number of seedlings from the dung of donkeys peaked in June, while the highest number of seedlings for goats occurred in July and August (Fig. 2a). Regarding the number of plant species dispersed in the seasonal course between March and August, the goat samples exhibited consistently fewer species than those of donkeys (Fig. 2b). The maximal number of plant species that germinated from the dung of donkeys was from the samples collected in June and July whereas for goats most plant species germinated from the dung samples of July.

**Table 3:** GLMM on seasonal influences on the plant dispersal (seedlings and species number) by donkeys and goats with estimates, standard error (SE) and p value

	Estimate	SE	p value
Seedlings number			
Intercept	0.522	0.305	0.086.
Goat	− 1.848	0.554	< 0.001***
August	1.425	0.494	0.003**
July	1.784	0.369	< 0.001***
June	2.177	0.398	< 0.001***
March	0.159	0.823	0.846
May	0.480	0.428	0.262
Goat:August	2.976	0.786	< 0.001***
Goat:July	2.619	0.645	< 0.001***
Goat:June	1.323	0.681	0.051
Goat:March	− 13.583	147.801	0.926
Goat:May	1.473	0.712	0.038*
Species number			
Intercept	0.145	0.211	0.490
Goat	− 1.427	0.453	0.001**
August	1.283	0.263	< 0.001***
July	1.570	0.217	< 0.001***
June	1.627	0.224	< 0.001***
March	0.423	0.492	0.389
May	0.483	0.275	0.078
Goat:August	1.119	0.524	0.032*
Goat:July	1.263	0.471	0.007**
Goat:June	0.691	0.487	0.156
Goat:March	− 14.238	228.973	0.950
Goat:May	0.780	0.551	0.156

p value with: *** = < 0.001; ** = < 0.01; * = < 0.05;. = < 0.1; n.s. = non-significant

Diagnostic species of the main vegetation types (Additional file 3), open grassland (GRA) and temporarily wet grassland (TWG), are correlated with the species germinated in the dung of donkeys (GRA: r_s_ = 0.40, p = < 0.001; TWG: r_s_ = 0.65, p = < 0.001; Additional file 4), whereas species emerged from the dung of goats are correlated with open grassland (GRA: r_s_ = 0.39, p = < 0.001), temporarily wet grassland (TWG: r_s_ = 0.42, p = < 0.001) and, rather weakly, with the semi-open maquis to preforest formations of *Juniperus phoenicea* (JUN: r_s_ = 0.21, p = 0.047) and the *Pinus pinea* forest (PIN: r_s_ = 0.22, p = 0.035).

## Discussion

We show clear differences between donkeys and goats in their potential and contribution as endozoochorous seed dispersers to the vegetation dynamics of a semi-natural island ecosystem in the Mediterranean. Donkeys and goats have different patterns of dispersal of plant functional types. In general, goats disperse a higher number of diaspores of all plant functional types except grasses. Shrubs and leguminous forbs are among the most dispersed functional types by goats. Donkeys, in contrast, disperse high numbers of grasses. Both, donkeys and goats, disperse the highest number of seedlings of the functional type sedges and rushes with a particularly high occurrence of Juncaceae which produce a high number of small sized seeds following the assumption that plants producing small seeds entail a high number of seeds [55, 56]. Bruun and Poschlod [57] reported that the number of seeds plays an important role in the plant dispersal ability by making more seeds available for dispersal. Furthermore, previous studies detected that small seeds are more likely to germinate from dung [58–60] and thus, it was suggested that the small size of seeds might be an essential characteristic for the survival of ingestion and gut passage [12, 61]. In addition, the type of digestive system might explain differences in the seed dispersal patterns between donkeys (hindgut fermenters) and goats (ruminants). Dispersal through endozoochory on one hand is affected by the survival of the seeds in the digestive tract [62] and on the other hand by the feeding habits [63]. The resulting dispersal spectrum from our experiment reflects the feeding habits of donkeys and goats. Donkeys prefer monocotyledons like grasses while the proportion of these in the fodder plants of goats is lower [42, 64]. Goats graze on shrubs, forbs and to a lesser extent on grasses [39, 65]. Even leaves and twigs of maquis shrubs and trees that generally exhibit a poor nutritional quality and comprise secondary metabolites (e.g. tannins, terpenes and volatile oils) like *Pistacia lentiscus*, *Juniperus phoenicae* and *Quercus ilex* are reported to be eaten by goats [66, 67]. This and the fact that goats are agile animals which can climb rocks and trees or use a bipedal posture to feed indicate the tendency of goats to forage in higher vegetation strata [68] and therefore they can disperse plant species that are less accessible for other grazers like donkeys. Our results corroborate this by showing divergent dispersal patterns of donkeys and goats for plants with different growth height. Donkeys disperse higher numbers of plants with shorter height, especially with growth heights between 10 and 40 cm. In contrast, goats mainly contribute to the dispersal of plants with greater heights (e.g. 80–100 cm) but also plants of the lowest height category (0–10 cm).

These patterns are reflected in the vegetation types that were predicted by the diagnostic species dispersed by donkeys and goats. Besides the potential vegetation of origin of the species germinated in the dung of donkeys and goats, the correlation of the diagnostic species of the main vegetation types with the endozoochorously dispersed species also provide information about the dispersal of character species of the respective vegetation type and thus of the vegetation type itself. Both, donkeys and goats, feed on and disperse species of the early-successional vegetation types open grassland (most relevant diagnostic species dispersed: *Phalaris minor*, *Astragalus pelecinus*) and temporarily wet grassland (most relevant diagnostic species dispersed: *Hordeum marinum*, *Mentha pulegium*). Both are characterized by a low growth and diagnostic species of the following plant functional types, grasses and leguminous forbs. However, temporarily wet grasslands can be distinguished by the occurrence of sedges and rushes. These vegetation types take a major part of the whole area of the island of which, open grassland comprises about 20% and temporarily wet grassland only ca. 2% [49]. Goats, in addition, feed on and disperse diagnostic species of the semi-open maquis to preforest formations of *Juniperus phoenicea* and the *Pinus pinea* forest, which both involve plants of diverse functional types and different growth heights. However, the diagnostic species of the vegetation type dominated by *Juniperus phoenicea* is herbaceous (most relevant diagnostic species dispersed: *Chenopodium murale*). Nevertheless, it indicates that goats might forage in this late-successional vegetation type and randomly disperse plant species of it. On the island of Asinara, the vegetation dominated by *Juniperus phoenicea* constitutes solely < 1% of the whole area and, for instance, the highly valuable coastal dune formations corresponding to the priority Natura 2000 habitat with code 2250 [69, 70] are rare and floristically impoverished [36]. As goats are indicated to feed in *Juniperus* formations and disperse diagnostic species, they also might enrich this vegetation type through their endozoochorous seed input. Other conservation relevant vegetation types like the coastal vegetation, which, for example, includes the endangered and to Sardinia endemic species *Centaurea horrida* [36, 71] did not show strong evidence that donkeys or goats feed and disperse its characteristic plant species. This vegetation type is highly restricted to the rocky coastal areas and threatened by overgrazing as well as abandonment of grazing activities leading to succession and thus competition [71, 72]. The *Pinus pinea* forest (most relevant diagnostic species dispersed: *Rubus ulmifolius*), which is also a late-successional habitat and constitutes solely < 0.5% of the whole area of the island and exhibits a rather low species richness, might also be enhanced in its species richness through the endozoochorous seed dispersal by goats. The impact of endozoochorous seed input on the vegetation and also on the seed bank can play an important role in colonization processes and at small scales [9, 15] at which the defecation pattern and the content of seeds in the dung are determining [9].

However, goats instead of a positive effect can also have a detrimental effect on the biodiversity especially under a non-adequate management and particularly in sensitive ecosystems like islands [33, 40, 41]. Grazing by goats and cattle was shown to inhibit the expansion of trees and woody vegetation [73]. The vegetation can even be irreversibly changed, which is often the consequence of too high numbers of grazing animals [74]. Plant diversity depends on grazing intensity, though some studies have shown that in Mediterranean semi-arid habitats low intensity of grazing tends to foster a low species diversity in the vegetation [23, 75, 76]. This might go back to the long history of grazing in the Mediterranean, thus wild and domestic grazers have shaped vast parts of the vegetation, rangelands, for example, comprise a vegetation mosaic that ranges from herbaceous vegetation, to scrubland and woody vegetation [75] or silvopastoral systems [77, 78].

Seasonal differences in the endozoochorous dispersal capacities of donkeys and goats show that donkeys in general disperse more species than goats and have an earlier dispersal peak. This might be due to the differing dispersal patterns of plant functional types and their divergent phenology. Phenology most likely is also the reason why species of scrubland and forest, like fleshy-fruited plants, are underrepresented. There is a peak of these species fruiting in autumn [79] which could not be recorded within this study. However, the majority of fleshy-fruited plant species in the Mediterranean are dispersed by birds or carnivorous mammals [80–82].

Our results point out differences in the dispersal capacity of donkeys and goats and thus indicate complementing seed input in a semi-natural island ecosystem. This corroborates the importance of the dispersal activities of both animal species. Removing an animal species completely from the island might lead to considerable changes in the vegetation dynamics of the island. Besides affecting the composition and structure of the vegetation, this abandonment might also change light conditions as well as physical and chemical characteristics of the soil [83]. Grazing animals are ecosystem engineers influencing the landscape dynamics and a potential conservation measure [84]. Supporting the importance of traditional land management, a directed and controlled transhumance of goats might be an option to integrate the benefits of goats to maintain and enrich the island ecosystem.

## Conclusions

Overall, our results show that donkeys and goats are complementing each other in their endozoochorous seed dispersal potential. This emphasizes the importance of both grazing animals for the vegetation dynamics of the semi-natural island ecosystem of Asinara. Nevertheless, besides the positive effects of goats, stocking rates and the extent of the degradation through them should be taken into consideration (e.g. over-grazing, soil damage and preventing regeneration).

## Additional files

**Additional file 1.** Digital mapping of the vegetation types of the island of Asinara. Colours represent the vegetation types.

**Additional file 2.** Plant species germinated in the dung samples of donkey and goat, their plant functional type and abundance in the dung samples of donkeys and goats of the Asinara National Park (Sardinia).

**Additional file 3.** Diagnostic species of the dominant vegetation types germinated in the dung samples of donkey and goat of the Asinara National Park (Sardinia). Phi coefficients are the measure of fidelity and p values are derived from a Monte Carlo permutation test.

**Additional file 4.** Correlations between number of germinated diagnostic species of the main vegetation types of Asinara island with number of viable species in the dung of donkeys and goats with r_s_ and p value.
