# Supplementary material for: Complementing endozoochorous seed dispersal patterns by donkeys and goats in a semi-natural island ecosystem
Source: BMC Ecol. 2017 Dec 19;17:42. doi: 10.1186/s12898-017-0148-6 (PMC5738203; doi:10.1186/s12898-017-0148-6)

Supplementary material

Complementing endozoochorous seed dispersal patterns by donkeys and goats in a semi-natural island ecosystem

Julia T. Treitler, Tim Drissen, Robin Stadtmann, Stefan Zerbe, Jasmin Mantilla-Contreras

Additional file 1 Digital mapping of the vegetation types of the island of Asinara. Colours represent the vegetation types

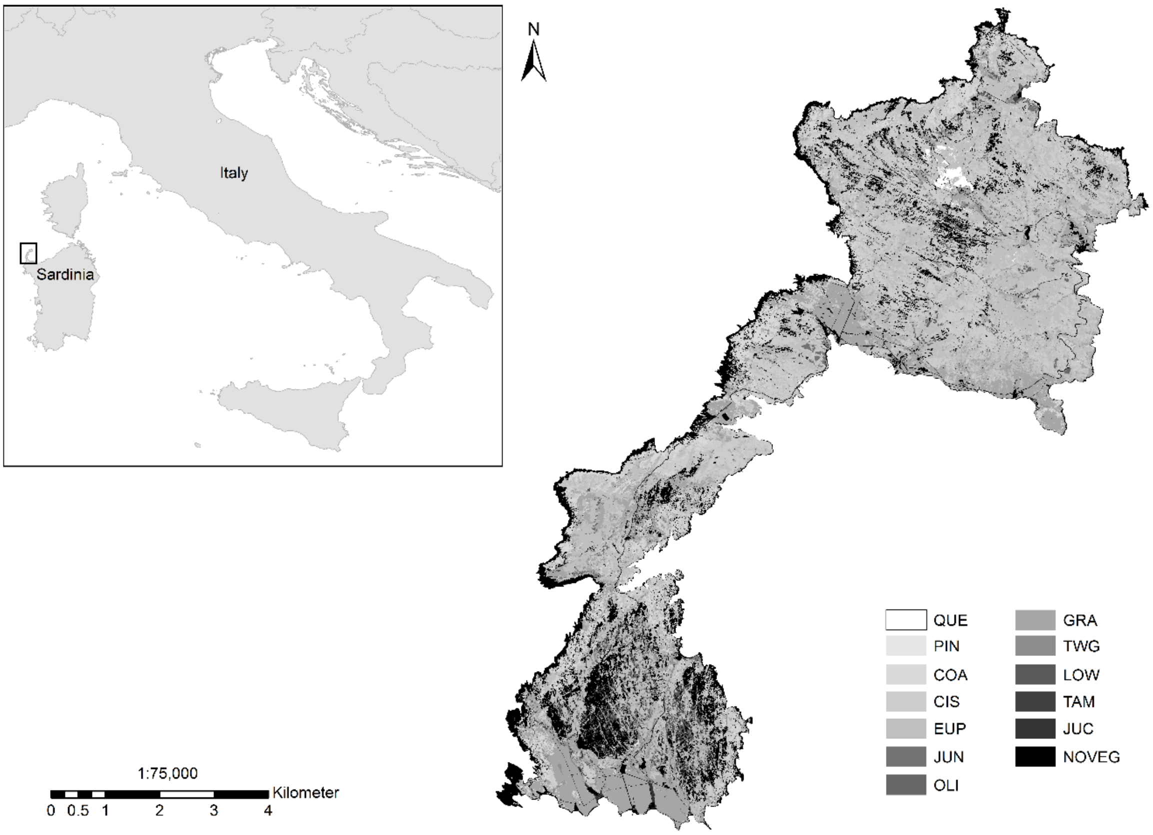

Supplement: Supplementary file 1 — Additional file 1. Digital mapping of the vegetation types of the island of Asinara. Colours represent the vegetation types. [file 12898_2017_148_MOESM1_ESM.pdf]
