# Supplementary material for: Complementing endozoochorous seed dispersal patterns by donkeys and goats in a semi-natural island ecosystem
Source: BMC Ecol. 2017 Dec 19;17:42. doi: 10.1186/s12898-017-0148-6 (PMC5738203; doi:10.1186/s12898-017-0148-6)
Supplement: Supplementary file 2 — Additional file 2. Plant species germinated in the dung samples of donkey and goat, their plant functional type and abundance in the dung samples of donkeys and goats of the Asinara National Park (Sardinia). [file 12898_2017_148_MOESM2_ESM.pdf]

Supplementary material

Complementing endozoochorous seed dispersal patterns by donkeys and goats in a semi-natural island ecosystem

Julia T. Treitler, Tim Drissen, Robin Stadtmann, Stefan Zerbe, Jasmin Mantilla-Contreras

Additional file 2 Plant species germinated in the dung samples of donkey and goat, their plant functional type and abundance in the dung samples of donkeys and goats of the Asinara National Park (Sardinia)

Family	Species	Plant functional type	donkey	goat
Amaranthaceae	*Chenopodium murale* L.	forb	0	10
Apiaceae	*Daucus carota* L. s.l.	forb	1	1
Apiaceae	*Torilis nodosa* (L.) Gaertn.	forb	1	7
Asteraceae	*Anthemis arvensis* L. s.l.	forb	2	22
Asteraceae	*Bellis perennis* L.	forb	1	0
Asteraceae	*Calendula arvensis* L.	forb	1	3
Asteraceae	*Chamaemelum fuscatum* (Brot.) Vasc.	forb	2	5
Asteraceae	*Crepis foetida* L.	forb	0	1
Asteraceae	*Filago gallica* L.	forb	0	1
Asteraceae	*Glebionis coronaria* (L.) Spach	forb	0	1
Asteraceae	*Helichrysum italicum* (Roth) Don subsp. *microphyllum* (Willd.) Nyman	shrub	0	1
Asteraceae	*Senecio vulgaris* L.	forb	1	0
Asteraceae	*Sonchus asper* (L.) Hill s.l.	forb	1	0
Brassicaceae	*Sisymbrium officinale* (l.) Scop.	forb	1	2
Caprifoliaceae	*Valerianella microcarpa* Loisel.	forb	0	1
Caryophyllaceae	Caryophyllaceae indet.	forb	1	0
Caryophyllaceae	*Cerastium glomeratum* Thuill.	forb	4	1
Caryophyllaceae	*Moenchia erecta* (L.) P. Gaertn., B. Mey. & Scherb. s.l.	forb	1	0
Caryophyllaceae	*Paronychia echinulata* Chater	forb	7	6
Caryophyllaceae	*Polycarpon tetraphyllum* (L.) L. s.l.	forb	1	0
Caryophyllaceae	*Sagina maritima* G. Don	forb	6	2
Caryophyllaceae	*Silene gallica* L.	forb	6	4
Caryophyllaceae	*Silene laeta* (Aiton) Godr.	forb	2	3
Caryophyllaceae	*Spergularia salina* J. & C. Presl	forb	4	1
Caryophyllaceae	*Stellaria media* (L.) Vill. s.l.	forb	2	0
Cistaceae	*Cistus monspeliensis* L.	shrub	2	8
Crassulaceae	*Phedimus stellatus* (L.) Raf.	forb	0	15
Crassulaceae	*Sedum rubens* L.	forb	2	2
Cyperaceae	*Carex divulsa* Stokes	sedges & rushes	1	0
Cyperaceae	*Carex microcarpa* Bertol. Ex Moris	sedges & rushes	2	0
Cyperaceae	*Carex* sp.	sedges & rushes	6	3
Cyperaceae	*Cyperus longus* L.	sedges & rushes	1	4

Cyperaceae	*Isolepis cernua* (Vahl) Roem. & Schult.	sedges & rushes	10	0
Fabaceae	*Astragalus pelecinus* (L.) Bameby	leguminous forb	1	1
Fabaceae	*Calicotome villosa* (Poir.) Link	shrub	0	4
Fabaceae	Fabaceae indet.	leguminous forb	0	2
Fabaceae	*Lotus angustissimus* L.	leguminous forb	0	1
Fabaceae	*Medicago arabica* (L.) Huds.	leguminous forb	0	1
Fabaceae	*Medicago polymorpha* L.	leguminous forb	1	1
Fabaceae	*Ornithopus pinnatus* (Mill.) Druce	leguminous forb	1	0
Fabaceae	*Trifolium angustifolium* L.	leguminous forb	0	7
Fabaceae	*Trifolium arvense* L. s.l.	leguminous forb	1	0
Fabaceae	*Trifolium campestre* Schreb.	leguminous forb	12	18
Fabaceae	*Trifolium cherleri* L.	leguminous forb	9	7
Fabaceae	*Trifolium glomeratum* L.	leguminous forb	31	45
Fabaceae	*Trifolium lappaceum* L.	leguminous forb	1	0
Fabaceae	*Trifolium ligusticum* Loisel.	leguminous forb	0	1
Fabaceae	*Trifolium nigrescens* Viv. s.l.	leguminous forb	2	6
Fabaceae	*Trifolium resupinatum* L.	leguminous forb	2	4
Fabaceae	*Trifolium scabrum* L.	leguminous forb	12	15
Fabaceae	*Trifolium subterraneum* L. s.l.	leguminous forb	1	0
Fabaceae	*Trifolium suffocatum* L.	leguminous forb	2	0
Fabaceae	*Vicia sativa* L. s.l.	leguminous forb	0	1
Frankeniaceae	*Frankenia laevis* L.	shrub	0	4
Gentianaceae	*Centaurium pulchellum* (Sw.) Druce	forb	0	1
Geraniaceae	*Geranium robertianum* L.	forb	0	5
Juncaceae	*Juncus acutus* L.	sedges&rushes	7	1151
Juncaceae	*Juncus articulatus* L.	sedges&rushes	1	1
Juncaceae	*Juncus bufonius* L. and *J. hybridus* Brot. species group	sedges&rushes	6	0
Juncaceae	*Juncus bufonius* L.	sedges&rushes	70	15
Juncaceae	*Juncus effusus* L.	sedges&rushes	0	1
Juncaceae	*Juncus hybridus* Brot.	sedges&rushes	40	12
Juncaceae	*Juncus* sp.	sedges&rushes	66	807
Lamiaceae	*Mentha pulegium* L.	forb	14	0
Onagraceae	*Epilobium tetragonum* L. s.l.	forb	0	10
Orobanchaceae	*Bartsia trixago* L.	forb	1	0
Plantaginaceae	*Plantago bellardii* All.	forb	1	3
Plantaginaceae	*Plantago coronopus* L.	forb	23	39
Plantaginaceae	*Plantago lagopus* L.	forb	16	16
Plantaginaceae	*Plantago lanceolata* L.	forb	0	3
Poaceae	*Agrostis pourretii* Willd.	grasses	3	1
Poaceae	*Agrostis stolonifera* L.	grasses	1	1
Poaceae	*Aira caryophyllea* L. s.l.	grasses	1	0
Poaceae	*Aira elegantissima* Schur	grasses	1	0
Poaceae	*Anthoxanthum aristatum* Boiss. s.l.	grasses	7	3
Poaceae	*Brachypodium retusum* (Pers.) P. Beauv.	grasses	2	0
Poaceae	*Briza maxima* L.	grasses	3	1

Poaceae	*Briza* sp.	grasses	9	0
Poaceae	*Bromus hordeaceus* L. s.l.	grasses	1	0
Poaceae	*Catapodium balearicum* (Willk.) H. Scholz	grasses	4	1
Poaceae	*Catapodium rigidum* (L.) C.E. Hubb. Ex Dony s.l.	grasses	0	1
Poaceae	*Cynodon dactylon* (L.) Pers.	grasses	7	0
Poaceae	*Cynosurus echinatus* L.	grasses	18	2
Poaceae	*Dactylis glomerata* L. s.l.	grasses	1	0
Poaceae	*Gastridium ventricosum* (Gouan) Schinz & Thell.	grasses	62	3
Poaceae	*Hainardia cylindrica* (Willd.) Greuter	grasses	1	0
Poaceae	*Hordeum marinum* Huds. s.l.	grasses	1	0
Poaceae	*Lagurus ovatus* L. s.l.	grasses	1	0
Poaceae	*Lolium perenne* L.	grasses	16	0
Poaceae	*Lolium rigidum* Gaudin s.l.	grasses	19	0
Poaceae	*Lolium* sp.	grasses	7	0
Poaceae	*Parapholis strigosa* (Dumort.) C.E. Hubb.	grasses	1	0
Poaceae	*Phalaris minor* Retz.	grasses	1	0
Poaceae	*Piptatherum miliaceum* (L.) Coss. s.l.	grasses	3	0
Poaceae	*Poa annua* L.	grasses	14	4
Poaceae	*Poa trivialis* L.	grasses	1	2
Poaceae	Poacea indet.	grasses	5	0
Poaceae	*Polypogon maritimus* Willd.	grasses	13	1
Poaceae	*Polypogon monspeliensis* (L.) Desf.	grasses	3	9
Poaceae	*Polypogon viridis* (Gouan) Breistr.	grasses	1	0
Poaceae	*Rostraria cristata* (L.) Tzvelev s.l.	grasses	2	1
Poaceae	*Vulpia ligustica* (All.) Link	grasses	1	0
Poaceae	*Vulpia myuros* (L.) C.C. Gmel.	grasses	1	0
Polygonaceae	*Rumex bucephalophorus* L. s.l.	forb	1	1
Polygonaceae	*Rumex crispus* L.	forb	1	0
Primulaceae	*Anagallis arvensis* L. s.l.	forb	4	5
Primulaceae	*Samolus valerandi* L.	forb	2	3
Rosaceae	*Rubus ulmifolius* Schott	shrub	0	64
Rubiaceae	*Sherardia arvensis* L.	forb	4	0
Rubiaceae	*Theligonum cynocrambe* L.	forb	1	1
Solanaceae	*Solanum nigrum* L.	forb	3	2
Solanaceae	*Solanum villosum* Mill. s.l.	forb	1	0
Urticaceae	*Parietaria judaica* L.	forb	0	3
			618	2395
