# Supplementary material for: Complementing endozoochorous seed dispersal patterns by donkeys and goats in a semi-natural island ecosystem
Source: BMC Ecol. 2017 Dec 19;17:42. doi: 10.1186/s12898-017-0148-6 (PMC5738203; doi:10.1186/s12898-017-0148-6)
Supplement: Supplementary file 3 — Additional file 3. Diagnostic species of the dominant vegetation types germinated in the dung samples of donkey and goat of the Asinara National Park (Sardinia). Phi coefficients are the measure of fidelity and p values are derived from a Monte Carlo permutation test. [file 12898_2017_148_MOESM3_ESM.pdf]

Supplementary material

Complementing endozoochorous seed dispersal patterns by donkeys and goats in a semi-natural island ecosystem

Julia T. Treitler, Tim Drissen, Robin Stadtmann, Stefan Zerbe, Jasmin Mantilla-Contreras

Additional file 3 Diagnostic species of the dominant vegetation types germinated in the dung samples of donkey and goat of the Asinara National Park (Sardinia). Phi coefficients are the measure of fidelity and p values are derived from a Monte Carlo permutation test

Vegetation type	Species	Phi	Phi Max	Vegetation type	p value	
COA	*Helichrysum italicum* subsp. *microphyllum*	0.766	0.766	COA	≤0.001	***
	Dactylis glomerata	0.519	0.519	COA	≤0.001	***
	Rumex bucephalophorus	0.502	0.502	COA	≤0.001	***
CIS	*Valerianella microcarpa*	0.555	0.555	CIS	0.0236	*
	Cistus monspeliensis	0.549	0.549	CIS	≤0.001	***
	Trifolium arvense	0.547	0.547	CIS	0.0088	**
EUP	-					
GRA	*Phalaris minor*	0.647	0.647	GRA	≤0.001	***
	Astragalus pelecinus	0.612	0.612	GRA	0.0024	**
	Trifolium nigrescens	0.574	0.635	TWG	≤0.001	***
	Vulpia ligustica	0.571	0.571	GRA	0.0034	**
	Rostraria cristata	0.543	0.543	GRA	0.0036	**
TWG	*Hordeum marinum*	0.883	0.883	TWG	≤0.001	***
	Mentha pulegium	0.756	0.756	TWG	≤0.001	***
	Juncus bufonius	0.728	0.728	TWG	≤0.001	***
	Silene laeta	0.728	0.728	TWG	≤0.001	***
	Trifolium nigrescens	0.635	0.635	TWG	≤0.001	***
	Juncus hybridus	0.632	0.632	TWG	≤0.001	***
	Agrostis pourettii	0.613	0.613	TWG	≤0.001	***
	Cyperus longus	0.610	0.610	TWG	0.006	**
	Trifolium resupinatum	0.586	0.586	TWG	≤0.001	***
	Lotus angustissimus	0.547	0.547	TWG	0.0068	**
	Polypogon monspeliensis	0.525	0.525	TWG	0.0158	*
	Chamaemelum fuscatum	0.520	0.520	TWG	≤0.001	***
JUN	*Chenopodium murale*	0.611	0.611	JUN	0.0032	**
OLI	*Solanum nigrum*	0.558	0.558	OLI	0.004	**
PIN	*Rubus ulmifolius*	0.696	0.696	PIN	0.0022	**
	Catapodium rigidum	0.555	0.579	QUE	≤0.001	***
QUE	*Carex divulsa*	0.756	0.756	QUE	≤0.001	***
	Poa trivialis	0.736	0.736	QUE	≤0.001	***
	Rubus ulmifolius	0.583	0.696	PIN	0.0022	**
	Catapodium rigidum	0.579	0.579	QUE	≤0.001	***
