# Supplementary material for: Complementing endozoochorous seed dispersal patterns by donkeys and goats in a semi-natural island ecosystem
Source: BMC Ecol. 2017 Dec 19;17:42. doi: 10.1186/s12898-017-0148-6 (PMC5738203; doi:10.1186/s12898-017-0148-6)
Supplement: Supplementary file 4 — Additional file 4. Correlations between number of germinated diagnostic species of the main vegetation types of Asinara island with number of viable species in the dung of donkeys and goats with rs and p value. [file 12898_2017_148_MOESM4_ESM.pdf]

Supplementary material

Complementing endozoochorous seed dispersal patterns by donkeys and goats in a semi-natural island ecosystem

Julia T. Treitler, Tim Drissen, Robin Stadtmann, Stefan Zerbe, Jasmin Mantilla-Contreras

Additional file 4 Correlations between number of germinated diagnostic species of the main vegetation types of Asinara island with number of viable species in the dung of donkeys and goats with r_s and p value

	Donkey			Goat		
	**r_s**	**p value**		**r_s**	**p value**	
COA	0.02	0.854		0.14	0.179	
CIS	0.01	0.916		0.04	0.726	
EUP	-	-		-	-	
GRA	0.40	<0.001	***	0.39	<0.001	***
TWG	0.65	<0.001	***	0.42	<0.001	***
JUN	-	-		0.21	0.047	*
OLI	0.03	0.810		0.14	0.189	
PIN	-	-		0.22	0.035	*
QUE	-0.07	0.524		0.14	0.179	

p value with: *** = <0.001; ** = <0.01; * = <0.05; . = <0.1; n.s. = non-significant
